# Combination of Classifiers Identifies Fungal-Specific Activation of Lysosome Genes in Human Monocytes

**DOI:** 10.3389/fmicb.2017.02366

**Published:** 2017-11-29

**Authors:** João P. Leonor Fernandes Saraiva, Cristina Zubiria-Barrera, Tilman E. Klassert, Maximilian J. Lautenbach, Markus Blaess, Ralf A. Claus, Hortense Slevogt, Rainer König

**Affiliations:** ^1^Network Modeling, Leibniz Institute for Natural Product Research and Infection Biology, Hans Knöll Institute, Jena, Germany; ^2^Integrated Research and Treatment Center, Center for Sepsis Control and Care, Jena University Hospital, Jena, Germany; ^3^Septomics Research Centre, Jena University Hospital, Jena, Germany

**Keywords:** classification, feature selection, gene expression, machine learning, SVM

## Abstract

Blood stream infections can be caused by several pathogens such as viruses, fungi and bacteria and can cause severe clinical complications including sepsis. Delivery of appropriate and quick treatment is mandatory. However, it requires a rapid identification of the invading pathogen. The current gold standard for pathogen identification relies on blood cultures and these methods require a long time to gain the needed diagnosis. The use of *in situ* experiments attempts to identify pathogen specific immune responses but these often lead to heterogeneous biomarkers due to the high variability in methods and materials used. Using gene expression profiles for machine learning is a developing approach to discriminate between types of infection, but also shows a high degree of inconsistency. To produce consistent gene signatures, capable of discriminating fungal from bacterial infection, we have employed Support Vector Machines (SVMs) based on Mixed Integer Linear Programming (MILP). Combining classifiers by joint optimization constraining them to the same set of discriminating features increased the consistency of our biomarker list independently of leukocyte-type or experimental setup. Our gene signature showed an enrichment of genes of the lysosome pathway which was not uncovered by the use of independent classifiers. Moreover, our results suggest that the lysosome genes are specifically induced in monocytes. Real time qPCR of the identified lysosome-related genes confirmed the distinct gene expression increase in monocytes during fungal infections. Concluding, our combined classifier approach presented increased consistency and was able to “unmask” signaling pathways of less-present immune cells in the used datasets.

## Introduction

A central goal of gene expression profiling studies is to identify key features that allow differentiation between specific clinical conditions of the patients of the corresponding samples (Ng et al., 2005). Several computational approaches have been developed to generate gene signatures with diagnostic potential: regression analyses, classification using decision trees, Random Forests and Support Vector Machines (SVMs) (Saeys et al., 2007). Especially the latter is a powerful method in the discovery-based approach (linking differential expression to a disease state) in the field of diagnostic biomarkers (Golub et al., 1999; Brown et al., 2000; Furey et al., 2000; Noble, 2004; Lee, 2007). One of the greatest advantages of SVMs is their implicit optimization for generalization by maximizing the separating hyperplane (McDermott et al., 2012; Batuwita and Palade, 2013). In the case of gene biomarker discovery for pathogen discrimination, the SVM can be employed to find the distinctive gene expression pattern that distinguishes best the type of infection (Brown et al., 2000). However, the generated gene signatures from independent studies usually do not present a high degree of consistency even if the same discrimination problem was addressed. We previously showed that combining classifiers using a Mixed Integer Linear Programming (MILP) improved consistency of gene signatures even if generated from quite diverse settings (Saraiva et al., 2016). The gene signature produced by Saraiva et al. accurately discriminated infected from non-infected samples with an average accuracy of 92% and was proposed as a generic host immune response toward infections due to the heterogeneity of the expression datasets in terms of immune cell stimulation. Gene set enrichment analysis revealed that two pathways were significantly enriched (Toll-like and Nod-like receptor signaling; Saraiva et al., 2016).

Whilst knowing if an individual is infected or not, it is essential to determine the type of the infection for the administration of the accurate therapy in the least amount of time (Bloos and Reinhart, 2014). Discriminating between fungal and bacterial infections is of vital importance, especially in the context of systemic infection. The current “gold standard” for pathogen identification relies on blood cultures which require several days for a result (Kirn and Weinstein, 2013).

In this study, we followed up on our previous investigations. The human immune system is complex and composed of many players. The innate immunity is the first line of defense against pathogens in the body. The ability to mount an adequate and effective innate immune response relies on the efficient and proper activation of, but not exclusively, both neutrophils and monocytes. Monocytes not only fight infections but can also differentiate into other immune cells such as macrophages and dendritic cells (DCs) which, in turn, are capable of phagocytic activity and provide the necessary stimulus to the adaptive immune system cells (Shi and Pamer, 2011; Lauvau et al., 2015). Monocytes express most of the pattern recognition receptors (PRRs) involved in fungal (Netea et al., 2008) and bacterial infections (Hessle et al., 2005), and studies have shown that the type of infection influences monocyte differentiation and, consequently, trigger different signaling cascades (Shi and Pamer, 2011). Monocytes take a pivotal role in the early pathogen recognition during candidiasis (Netea et al., 2008; Klassert et al., 2014; Ngo et al., 2014) and have been suggested to be the most effective type of innate immune cells in the killing of *C. albicans* (Netea et al., 2008).

Considering the ratio of the different immune cells we hypothesized that the effect on specific pathways of a less abundant type of immune cells could be “masked” by the overwhelming effect of more numerous leukocytes such as neutrophils or lymphocytes. Studies have shown that the expression of several genes is immune cell type-specific (Wong et al., 2011; Allantaz et al., 2012; Gardinassi et al., 2016; Petryszak et al., 2016). Other studies have also shown that gene overexpression can activate distinct molecular pathways depending on the cell population (Liu et al., 2015; Didonna et al., 2016). Cell-type specific gene expression studies have also shown that the relative proportion of each leukocyte type invariably has an impact on the global gene expression profile (Palmer et al., 2006). In the same study, the set of genes with the highest relative expression in lymphoid cells presented the lowest relative expression in whole blood (e.g., *CD3G, LEF1, TCF7, CD3D, MAL*, and *CD2*). In our study, we employed the combined classifier approach we develop earlier (Saraiva et al., 2016) on datasets of similar leukocyte compositions and aimed to determine if these similarities also present specific signaling pathways not uncovered by the generic approach on the immune response in our previous study.

## Methods

### Dataset assembly

The normalized gene expression data from two datasets (accession numbers: GSE42606 and GSE69723) was obtained via Gene Expression Omnibus (GEO) (www.ncbi.nlm.nih.gov/geo/) from the National Center for Biotechnology Information (NCBI) database. RNA-Seq data was retrieved from NCBI's Sequence Read Archive (SRA). A study performed by Klassert et al. (Klassert et al., 2017; Riege et al., 2017), and hereon identified as “Klassert,” generated RNA-Seq data (accession number SRP076532) which consisted of healthy human blood-derived monocytes stimulated with heat-killed *Aspergillus fumigatus* AF293, *Candida albicans* SC5314 yeast (both at a Multiplicity of infection (MOI) of 1), *Escherichia coli* serotype O18:K1:H7 (MOI of 10) or left untreated (control). Cells were stimulated for 3 and 6 h after which their RNA was extracted. On the raw reads a sequence quality analysis was performed using FastQC version 0.10.1 and a read trimming to 150 bp was performed using FASTX Toolkit 0.0.14 and adapter trimming using cutadapt version 1.3. Reads were mapped onto the reference genome GRCh38/hg38 from the UCSC server and counted for each gene across all samples using HTSeq-count. The read number per gene, total read number per sample and gene length was then used to calculate the Reads Per Kilobase of transcript per Million mapped reads (RPKM) values across all genes and samples. Genes with RPKM values of 0 across all samples were removed. Smeekens and co-workers (Smeekens et al., 2013) performed a study in which peripheral blood mononuclear cells (PBMCs), isolated from blood of healthy human donors, were stimulated with heat-killed *C. albicans* UC820 (1 × 10^6^/mL), *Mycobacterium tuberculosis* (10 ng/mL) and LPS derived from *E. coli* (10 ng/mL). Cells grown in Roswell Park Memorial Institute Medium (RPMI) culture medium were used as controls (accession number GSE42606). Samples were taken at 4 and 24 h after infection. In this dataset, only the 4-h time point was considered for our studies since we were investigating the innate immune response in the acute phase. For future reference, this dataset will be identified as “Smeekens.” Transcriptomic data generated by Saraiva et al. (2016), and hereby identified as “Saraiva,” was generated by challenging healthy human blood-derived PBMCs with either heat-killed *C. albicans* MYA-3573 yeast (MOI of 2) or LPS derived from *E. coli* 0111:B4 (10 ηg/mL) (InvivoGen). Four samples were extracted 4 h post-infection. RNA was extracted using RNAEasy Kit Qiagen and quantity and quality of the total RNA was analyzed using a Nanodrop ND-1000 spectrophotometer (Thermo Fischer Scientific, USA) and a Tape Station 2200 (Agilent Technologies, USA). Lastly, transcriptional data of human blood isolated monocytes challenged with *A. fumigatus* conidia (MOI of 2) and LPS (10 ng/mL) was downloaded from the European Molecular Biology Laboratory (EMBL) ArrayExpress database (E-MEXP-1103) (http://www.ebi.ac.uk/arrayexpress/experiments/E-MEXP-1103/) and is hereby identified as “Mattingsdal.” A total of 5 and 6 samples were extracted 6 h post-challenge (*A. fumigatus* and LPS, respectively).

### Data preprocessing

Each dataset was controlled if prior normalization had been executed on the expression data. In the absence of normalization, the following was performed: RNA-Seq data was log2 transformed and a 1% quantile added onto all values, whilst microarray data was normalized by employing the functions “lumiN” and method “vsn” of the “lumi” R package (Du et al., 2008). Elimination of possible duplicate gene entries was carried out by use of the “avereps” function in the “limma” R package (Ritchie et al., 2015), which calculates the mean expression values for duplicate entries. Finally, z-scores were calculated for each gene. The gene list, to be used for feature selection and classification, consisted of the intersection of the gene lists from all datasets and amounted to 1,567 genes.

### Classification

In each dataset, the samples were grouped into either fungal (class 1) or bacterial (class 2). The number of samples in each dataset for each analysis is shown in Table 1. For classification and feature selection, we employed Support Vector Machines (SVMs) implemented with Mixed Integer Linear Programming as previously described (Saraiva et al., 2016) and with the same parameters (number of cross-validations (runs) and number of features (genes) to be selected in each cross-validation) as explained in the following (full implementation procedure in the Text S1). This process was done for both single and combined classifiers to compare both approaches. Briefly, during each cross-validation, SVMs were constrained to n = 30 features (genes) and they selected these with which they best discriminated between fungal and bacterial infected samples on the training data. Two thirds of the samples were randomly selected for training whilst one third was used for testing. This procedure was repeated 100 times. To remove the possible imbalance between classes, a stratified approach was employed in which the maximum number of samples to be used in each class was determined by the class with the least number of samples.

**Table 1:** Number of samples in each dataset divided into fungal and bacterial class.

**Dataset**	**Cell type**	**Fungal class**	**Bacterial class**
Smeekens	PBMC	24	49
Mattingsdal	Monocytes	5	6
Klassert	Monocytes	18	9
Saraiva	PBMC	4	4

To remove less frequently selected genes, further filtering of the gene lists was performed. Genes not selected in at least 20 runs (out of 100) in each classifier (both single and combined) were removed. The resulting gene lists were then merged into their respective group (either single or combined approach). Ascertaining the functional overview of the refined gene lists was achieved by performing literature analysis as well as using the functional annotation tools of the Database for Annotation, Visualization and Integrated Discovery (DAVID, version 6.7, https://david.ncifcrf.gov/home.jsp (Huang da et al., 2009) using *Homo sapiens* background. The full workflow is depicted in Figure S1.

### Differential gene expression analysis

In each dataset we calculated differentially expressed genes using Student's *t*-tests with multiple testing correction (Benjamini-Hochberg method, Benjamini and Hochberg, 1995). Genes were regarded as differentially expressed if their adjusted *p*-value was below 0.05. Intersection of differentially expressed genes was performed for all datasets and according to leukocyte composition (all datasets, PBMC specific and monocyte specific). Gene set enrichment analysis, for each list, was carried out as stated above.

## Experimental validation

### Monocyte isolation

Buffy coats of healthy male donors for cell isolation were kindly provided by Dagmar Barz in anonymized form (Institute of Transfusional Medicine of the Jena University Hospital). Human monocytes were isolated from 50 ml buffy coats of four healthy male donors as previously described (Müller et al., 2017). Briefly, ficoll-density gradient centrifugation was used to isolate first peripheral blood mononuclear cells (PBMCs). After restoring the osmolarity of the cells with 0.45% NaCl, remaining erythrocytes were lysed using a hypotonic buffer. Where needed, 5 × 10^6^ PBMCs were seeded in 6-well plates (VWR International, Germany) and allowed to equilibrate for 1 h at 37°C 5% CO_2._ From the remaining PBMCs, monocytes were then isolated using quadro-MACS (Miltenyi Biotec, UK) by labeling the non-monocytic cells with a cocktail of Biotin-conjugated antibodies and Anti-Biotin Microbeads (Monocyte Isolation Kit II, Miltenyi Biotec, UK). Cell viability of >98% was assayed by Trypan blue staining. Monocyte concentration was adjusted to 2.5 × 10^6^ cells/ml in RPMI 1640 GlutaMAX medium (Gibco, UK) supplemented with 10% fetal bovine serum (FBS, Biochrom, Germany) and 1% Penicillin/Streptomycin (Thermo Fisher Scientific, USA), 5 × 10^6^ cells were seeded in 6-well plates (VWR International, Germany) and allowed to equilibrate for 1 h at 37°C 5% CO_2_.

### Preparation of fungi and bacteria

Overnight culture from *Escherichia coli* (isolate 018:K1:H7) in LB medium was washed twice in PBS and resuspended in 1 ml RPMI 1640 GlutaMAX medium (Gibco, UK) supplemented with 10% FBS (Biochrom, Germany) at a concentration of 5 × 10^8^ cfu/ml. *Aspergillus fumigatus (*AF293) was grown in Aspergillus Minimal Medium (AMM) Agar-plates for 6 days at 30°C. Conidiospores were harvested by rinsing the plates with sterile 0.05% Tween-20 (Sigma-Aldrich, Germany) and filtered through 70- and 30-μm pre-separation filters (Miltenyi Biotec, UK) to get rid of mycelium traces. Spores were washed twice in PBS and cell-concentration was adjusted to 10^7^ conidia/ml in RPMI 1640 GlutaMAX medium supplemented with 10% FBS. Conidia were then incubated at 37°C under shaking for 7 h until cells turned to germ tubes. Germlings were centrifuged and resuspended at 1 × 10^8^ cells/ml in RPMI 1640 GlutaMAX medium supplemented with 10% FBS. Overnight culture of *Candida albicans* (SC5314) in YPD medium was washed twice in PBS and cell concentration was adjusted to 5 × 10^7^ cfu/ml in RPMI 1640 GlutaMAX medium supplemented with 10% FBS.

### Monocyte stimulation assay

Pathogens were all heat-killed by incubation at 65°C for 30 min before infection. Monocytes were stimulated with heat-killed pathogens at a pathogen:host ratio of 10:1 for bacteria, 1:1 for *A. fumigatus* germ tubes and *C. albicans* yeasts. In addition, cells were stimulated with pathogen-derived cell wall components: LPS (50 ng/ml) and zymosan (1 μg/ml). After 3 h incubation at 37°C and 5% CO_2_, monocytes were lysed for RNA isolation. To analyse the expression level of the genes of interest, total RNA was extracted from 5 × 10^6^ Monocytes using the Qiagen RNeasy mini kit (Qiagen, Germany). Residual genomic DNA was removed by on-column incubation with DNaseI (Qiagen, Germany). A NanoDrop D-1000 Spectrophotometer (Thermo-Fisher Scientific, USA) was then used to assess the amount and quality of the isolated RNA samples. Complementary DNA (cDNA) was synthesized from 1.5 μg of RNA using the High Capacity cDNA Reverse Transcription Kit (Applied Biosystems, UK) following manufacturer's instructions. To detect the expression of the genes by PCR, specific primers for each target were designed using the online Primer-BLAST tool of the NCBI (http://www.ncbi.nlm.nih.gov/tools/primer-blast/). Possible secondary structures at the primer binding sites were taken into account by characterizing the nucleotide sequence of the regions of interest using the Mfold algorithm (Zuker, 2003). The sequences of all primers used for amplification are listed in Table S1. For quantification of the relative expression of each gene, we used a CAS-1200 pipetting robot (Qiagen) to set up the qPCR-reactions and a Corbett Rotor-Gene 6000 (Qiagen) as Real-Time qPCR apparatus. Each sample was analyzed in a total reaction volume of 20 μl containing 10 μl of 2 × SensiMix SYBR Master Mix (Bioline, UK) and 0.2 μM of each primer. The cycling conditions included an initial step of 95°C for 10 min followed by 40 cycles of 95°C for 15 s, 60°C for 20 s and 72°C for 20 s. For each experiment, an RT-negative sample was included as a control. Melting curve analysis and primer efficiency was used to confirm the specificity of the qPCR reactions. The relative expression of the target genes was analyzed using the Pfaffl method (Pfaffl et al., 2004; Rieu and Powers, 2009). To determine significant differences in the mRNA expression between different experimental conditions, the relative quantity (RQ) for each sample was calculated using the formula 1/E^Ct^, where E is the efficiency and Ct the threshold cycle. The RQ was then normalized to the housekeeping gene peptidylprolyl isomerase B (*PPIB*). The stability of the housekeeping gene was assessed using the BestKeeper algorithm (Pfaffl et al., 2004). The normalized RQ (NRQ) values were log_2_-transformed for further statistical analysis with GraphPad PRISM v7.02. Statistical analysis was performed using repeated measures one way ANOVA and Bonferroni correction.

## Results

Classification was performed on each individual dataset (“Klassert,” “Smeekens,” “Saraiva,” and “Mattingsdal”) using 100 randomly assigned training sets within a cross-validation scheme. A list of 30 genes was generated in each classification run which best discriminated samples infected with fungal from bacterial pathogens. Consistency of the gene signature was determined by calculating the pairwise overlap (POL) between cross-validations of each classifier. Briefly, the gene list (*n* = 30) of each cross-validation of one dataset was intersected with the gene list of each cross-validation of another, different dataset. This was done for each pair of single classifiers. To obtain better consistency (as we observed in our initial study identifying biomarkers for infection irrespective of the kind of infection, see Saraiva et al., 2016), we also combined classifiers of two datasets (e.g., Smeekens with Klassert) constrained to use the same feature selection. We intersected the 100 cross-validations between single classifiers, between single and combined classifiers and between only combined classifiers. An illustrative example of this procedure is depicted in Figure S2. The averaged POL of the 100 generated gene lists of single vs. single, single vs. combined and combined vs. combined classifiers returned values of 0.78 (1σ = 0.41), 1.09 (1σ = 0.48), and 1.64 (1σ = 0.49), respectively. The POL of combined vs. single already showed an increase in almost 40% when compared to single vs. single, increasing to more than 100% when calculating the POLs between combined classifiers.

Next, we aimed at determining which pathways were significantly enriched in both the single and combined classifier gene lists. In each classifier (single and combined approach), the genes not selected in at least 20% of the total number of runs (*k* = 100) were excluded from further analysis. A total of 175 and 164 genes, for single and combined classifiers, respectively, remained (Table S2). The enriched pathways of single and combined gene signatures are shown in Tables 2, 3, respectively. Interestingly, one enriched gene set of the combined classifier gene list was not present in that of the single classifier—Lysosome (KEGG pathway). Next, independent from the classifier results, for each dataset, differentially expressed genes in fungal vs. bacterial infected samples was calculated. The intersection of the differentially expressed genes across all datasets resulted in a list of 13 genes (*ST3GAL5, HMOX1, LGALS9, GLA, HAVCR2, TBC1D9, ACADVL, BCAR3, RHOU, MGAT2, CCL23, RGS1*, and *SPRY2*) and no enriched gene sets. Intersection of differentially expressed genes was performed not only for all datasets but now also based on the type of the immune cells to shape out the origin of these differences in gene expression.

**Table 2:** Enriched gene sets of the single classifier approach.

**Gene set**	***P*-value**
Chemokine signaling	2.3E-17
Cytokine-cytokine receptor interaction	8.6E-15
Toll-like receptor signaling	2.7E-5
Jak-STAT signaling	7.2E-4
Chronic myeloid leukemia	0.0011
Leukocyte transendothelial migration	0.011
Natural killer cell mediated cytotoxicity	0.192
B cell receptor signaling	0.031
Fc epsilon RI signaling	0.035
Intestinal immune network for IgA production	0.042

**Table 3:** Enriched gene sets of the combined classifier approach.

**Gene set**	***P*-value**
Toll-like receptor signaling	2.2E-4
Cytokine-cytokine receptor interaction	3.1E-4
Lysosome	0.014
Chemokine signaling	0.027
Jak-STAT signaling	0.042

As stated before, monocytes are vital players in the control of infection, by both promoting inflammation and differentiating into other immune cells. The processes that they influence, however, can be distinct to those of other more abundant immune cells such as lymphocytes and the expressed genes of monocytes may hence be “masked.” To elucidate this masking phenomenon, we calculated the differentially expressed genes of the datasets of the PBMCs (datasets Saraiva, Smeekens), and of the monocytes (Klassert, Mattingsdal) separately. Intersecting differentially expressed genes, both up and down regulated, of the datasets encompassing solely monocytes resulted in 720 genes, whilst the intersection of datasets comprised of PBMCs resulted in a list of 57 genes. The enriched gene sets, for PBMC-specific and monocyte-specific differentially expressed genes are shown in Table 4. The enriched gene sets in all groups suggested that genes coding for the lysosome were specifically induced by monocytes during a fungal challenge. To note, the combined classifier-originated gene list also showed an enrichment of genes coding for the lysosome (lysosome gene set in the following). Additionally, we intersected the differentially expressed and up-regulated genes (in fungal vs. bacterial) from the monocyte datasets (Klassert and Mattingsdal) and performed gene set enrichment tests. Only two pathways were significantly enriched—the lysosome and Toll-like receptor signaling (*P* = 3.2E-4 and 0.015, respectively). We believe that this strengthens our initial finding that cell type specific gene expression is still captured when combining classifiers, without the requirement of performing a cell type specific analysis beforehand. Performing gene set enrichment tests on differentially expressed genes from cell type specific datasets produced the same results.

**Table 4:** Enriched gene sets of PBMC-specific and monocyte-specific differentially expressed genes in fungal vs. bacterial infection (both up and down regulated).

**PBMC-specific**		**Monocyte-specific**	
**Gene set**	***P*-value**	**Gene set**	***P*-value**
Jak-STAT signaling	0.0011	Toll-like receptor signaling	2.5E-5
Toll-like receptor signaling	0.0035	NOD-like receptor	3.5E-5
Cytokine-cytokine receptor interaction	0.046	Hematopoietic cell lineage	2.4E-4
		Cytokine-cytokine receptor interaction	3.9E-4
		Chemokine signaling	0.0018
		Jak-STAT signaling	0.0035
		Lysosome	0.0044
		Cytosolic DNA-sensing	0.0049
		MAPK signaling	0.0054
		Adipocytokine signaling	0.016

Gene set enrichment was also performed on the gene list that resulted in the intersection of differentially expressed and up regulated genes considering only the datasets of stimulated PBMCs, and comprised of Jak-STAT signaling, cytokine-cytokine receptor interaction and toll-like receptor signaling (Table S3).

In summary, we identified a few, well selected, distinct gene sets being enriched in differentially expressed genes discriminating fungal from bacterial infection, and when elucidating gene sets specifically expressed in monocytes by our combined classifier approach and a monocyte specific analysis, the lysosome gene set came out to be highly enriched in discriminative genes. Hence, in the following, we focused on the lysosomal gene set.

### Experimental validation

We reproduced the experimental settings of the studies herein considered focussing on monocytes, and stimulated human monocytes with the respective pathogens. The validation of the expression profiles observed for monocytes in the RNA-Seq data was performed using quantitative RT-PCR. For this purpose, we first tested the stability of the housekeeping gene used (*PPIB*). Using the algorithm BestKeeper (Pfaffl et al., 2004), the expression stability (*std dev* ± *CP*) and coefficient of variation (CV) for the housekeeping gene was calculated for monocytes. On this basis, *PPIB* was proved as a highly stable housekeeping gene for relative expression analyses (*std dev* ± *CP* = 0.35; *CP* = 1.95 %).

### Lysosome-related genes

**Figure 1:**
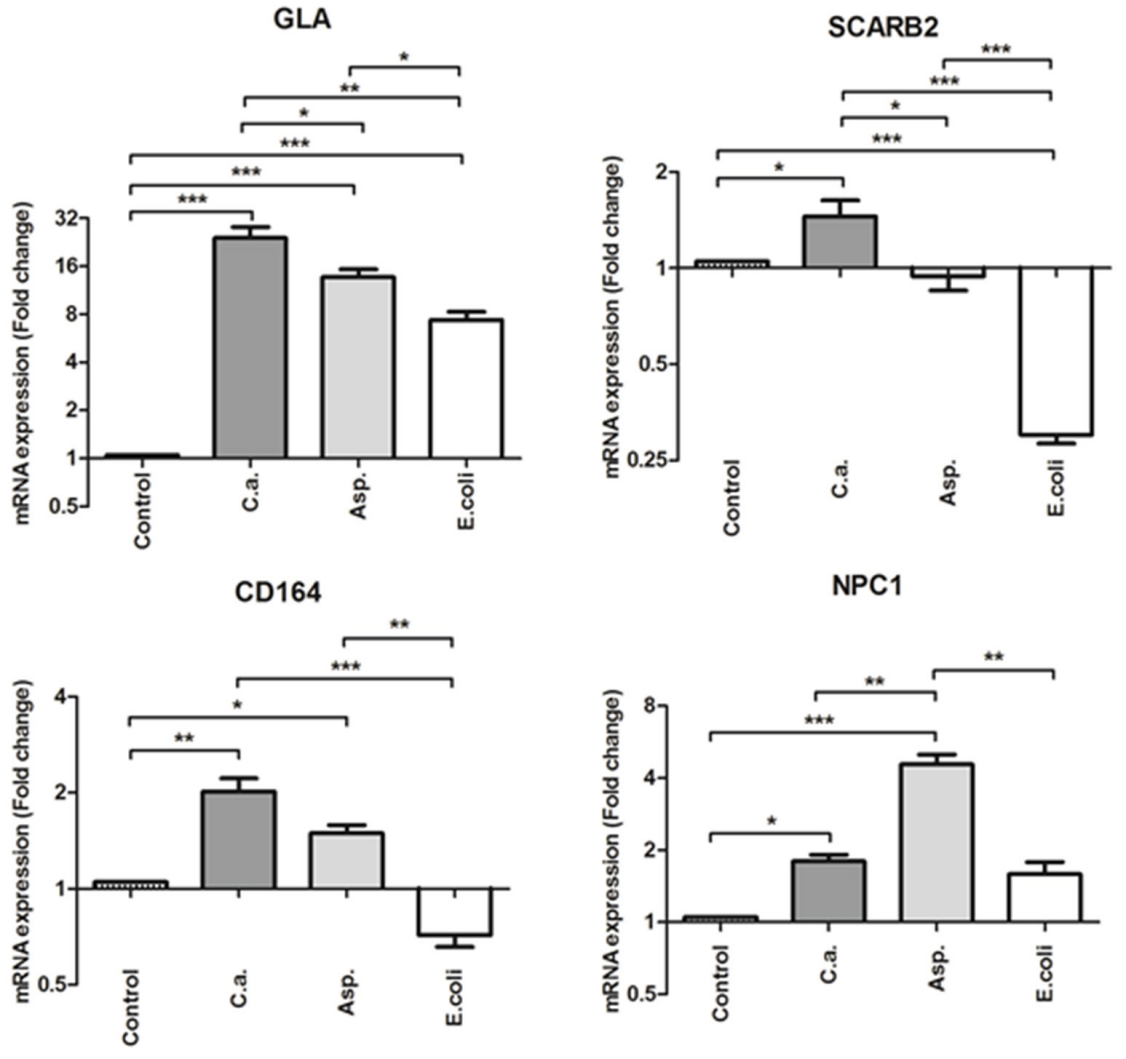
Relative mRNA expression of *GLA, SCARB2, CD164*, and *NPC1* after stimulation with *Candida albicans* (C.a.), *Aspergillus fumigatus* (Asp.) and *Escherichia coli* (*E. coli*). Data were obtained from four independent experiments, each performed with cells from different donors. Results are presented as mean ± SE of the fold change relative to the control (unstimulated cells) according to (Pfaffl et al., 2004). (Please see also: Rieu and Powers, 2009. Real-Time Quantitative RT-PCR: Design, Calculations, and Statistics. The Plant Cell; Vol. 21: 1031–1033. Shown is also the statistical significance after repeated measures One-Way ANOVA after multiple testing correction (Bonferroni) (^***^p < 0.001; ^**^p < 0.01; ^*^p < 0.05).

Based on the results obtained using our combined classifier approach, 4 lysosome-related genes were selected for validation by real time RT-qPCR analysis. These were the genes encoding for Galactosidase A (*GLA*), Scavenger receptor class B member 2 (*SCARB2*), Niemann-Pick disease, type C1 (*NPC1*) and the *CD164* molecule (*CD164*). The real-time RT-qPCR plots are shown in Figure 1 (The complete table of the RT-qPCR mean expression values across conditions and corresponding *p*-values are shown in Table S4). Almost all genes showed a significant increase in their expression when the fungi-stimulated group was compared to either the unstimulated controls and/or to the bacteria-challenged samples. *GLA* was significantly up-regulated by both fungal pathogens when compared to control and to *E. coli*-stimulated monocytes. *SCARB2* was up-regulated in a highly significant manner in *C. albicans*-stimulated monocytes when compared *to E. coli*-challenged monocytes. *SCARB2* also showed a significant increase in expression when compared to controls and *A. fumigatus*-challenged monocytes. In *E. coli* stimulated monocytes, *SCARB2* was significantly down-regulated when compared to controls. *NPC1* showed a significant increase in its expression in *A. fumigatus*-stimulated monocytes when compared to all other challenges. *C. albicans*-stimulated monocytes also showed significant increase of *NPC1* expression when compared to controls. Lastly, *CD164* was significantly up-regulated in both fungi when compared to *E. coli* and controls. In summary, we could validate the expression of the selected genes to be either specifically or significantly more up-regulated in monocytes stimulated by fungal pathogens when compared to monocytes stimulated by bacterial pathogens confirming them as potential biomarkers for fungal vs. bacterial induced systemic infection.

The fungi-specific pattern observed for lysosome-related genes in monocytes was less evident in PBMCs, as confirmed by an additional set of experiments in which monocytes and PBMCs from the same donors were stimulated in parallel with *C. albicans, A. fumigatus*, and *E. coli* (Figure S3). These results are in accordance with the microarray and RNA-Seq readouts from the different datasets analyzed (Monocytes vs PBMCs), and might explain why the lysosome-pathway was significantly enriched only in the monocyte datasets.

### Lysosome-unrelated genes

Our approach identified additional genes that showed a differential pattern in leukocytes upon fungal vs. bacterial infection but unrelated to the lysosome. These included the BAG family molecular chaperone regulator 3 (*BAG3*), the fatty acid binding protein 5 (*FABP5*), the peroxisome proliferator-activated receptor gamma (*PPARG*), the heme oxygenase 1 (*HMOX1*) and the C-C chemokine receptor type 1 (*CCR1*). Real-time qPCR of these genes showed a significant (*P* ≤ 0.05) increased expression in fungal stimulated monocytes when compared to all other stimuli. Except for *BAG3*, all other genes were downregulated after *E. coli* stimulation, reaching statistical significance for two of the genes (*HMOX1* and *CCR1*) (Figure S4).

## Discussion

Accurate identification of key features that allow for differentiation between specific clinical conditions represents an important challenge with diagnostic potential for the clinical daily practice. As shown in our previous study (Saraiva et al., 2016), the consistency of differential gene signatures increases substantially after combining classifiers when compared to single classifiers. The application of the combined classifier approach limits the impact of many variables that exist when comparing datasets such as the pathogen strain, laboratory settings, time of sample extraction and stimulated cell-type (e.g., PBMCs or whole blood), amongst others. This is particularly important when trying to generate a generic gene signature capable of discriminating infections irrespective of the immune cell type. In the present work, we validated our method on specific populations of immune cells, and demonstrated its ability to identify cell-specific signatures that were masked in mixed populations if using classifiers without combining the datasets. As observed in our results, combining classifiers for discrimination between fungal and bacterial infections in different leukocyte-compositions, such as PBMCs and monocytes, generated a gene signature enriched for several immune signaling pathways, among which the lysosome gene set was observed which turned out to be specific for monocytes. This was ascertained by the comparison of the enriched signaling pathways of differentially expressed genes in cultures of monocytes against PBMCs, both challenged with fungal and bacterial pathogens. We validated our results experimentally employing qPCR, analyzing a set of lysosome-related genes that were either selected by the combined classifier or uniquely differentially expressed in the monocyte challenged datasets. As shown, all the lysosome-related genes (*GLA, SCARB2, NPC1*, and *CD164*) exhibited a significant increase in their expression after fungal challenge when compared to bacterial stimulation, indicating a fungal-specific response by monocytes (Figure 1). Similar results were also obtained for other, non-lysosome related genes that were part of the fungal-specific signature and also these genes could be validated by qPCR (Figure S4). These genes included *BAG3, PPARG, FABP5, HMOX1*, and *CCR1*.

### Functional relevance of the differentially expressed lysosome-related genes

α-Galactosidase A (GLA) is a glycoside hydrolase enzyme encoded by the *GLA* gene. This enzyme hydrolyses the terminal α-galactosyl moieties (especially the α-1,6 linkage) of glycoproteins and glycolipids. Specifically, GLA is a lyososmal enzyme that degrades globotriaosylceramide (Gb3) to lactosylceramide, preventing its accumulation in this compartment (Darmoise et al., 2010). Deficiency of this enzyme (GLA) and accumulation of the glycolipid Gb3 in the lysosome of peripheral blood mononuclear cells (PBMCs) has been shown to contribute to diverse physiopathological alterations such as the continuous pro-oxidative and pro-inflammatory state of these cells (De Francesco et al., 2013). Moreover, a pro-inflammatory role of Gb3 could be demonstrated in that study, which was directly mediated by the TLR4-pro-inflammatory signaling pathway (De Francesco et al., 2013). *Candida albicans* yeast, among other fungi, binds to TLR4 that recognizes short linear O-bound mannan structures present in the fungal cell wall (Netea et al., 2008). Besides this, the GLA product lactosylceramide has been reported to be very abundant on plasma membranes of phagocytes, being involved in the phagocytosis, chemotaxis, and superoxide generation during fungal infection (Jimenez-Lucho et al., 1990; Iwabuchi et al., 2015). Our results show that *C. albicans* and *A. fumigatus* induce a significantly higher expression of the *GLA* gene than *E. coli*, suggesting the importance of this enzyme in monocytes during fungal infection. Among all the lysosome-related genes analyzed in this study, *GLA* showed the strongest up-regulation upon pathogen-challenge, particularly during fungal stimulation (24-fold for *C. albicans* and 14-fold for *A. fumigatus*). It might be speculated that GLA avoids the accumulation of the glycolipid Gb3 in the lysosome as an anti-inflammatory and protective mechanism in monocytes, which might be of special importance during fungal clearance. Moreover, the conversion of Gb3 to lactosylceramide, as a membrane microdomain of immune cells, may increase the phagocytosis and clearance of the fungi.

Scavenger receptor class B member 2 (*SCARB2*) is a gene whose encoded protein, the lysosomal integral membrane protein type-2 (LIMP-2/SCARB2), has been shown to be essential for the normal biogenesis and maintenance of lysosomes and endosomes (Gonzalez et al., 2014). As a lysosomal membrane protein, SCARB2 has been reported to act as an entry receptor for Enterovirus 71 (EV71) leading to its internalization to the lysosome (Yamayoshi et al., 2014). Other scavenger receptors, such as CD36 and SCARF1 (human homologs of the murine C03F11.3 and CED-1, respectively), have been shown to bind *C. neoformans* and *C. albicans* via ß-glucan structures, providing protection against these fungal pathogens in a mice model (Croze et al., 1989). Not much is known about the function of SCARB2 during fungal induced immune responses, but our results suggest that this scavenger receptor, like other similar members of this protein family, may play an important role in fungal recognition and internalization to the lysosome. Moreover, we analyzed whether the most common fungal and bacterial cell wall components (the fungal ß-glucan and the bacterial lipopolysaccharide, respectively) could explain the differential regulation of this gene by the different pathogens. *E. coli*-derived LPS resembled the downregulation of *SCARB2* already observed after stimulation with *E. coli* cells. In contrast, the fungal ß-glucan component seems to have no effect on the regulation of this gene (Figure S5). From these results we could conclude that the bacterial liposaccharide seems to be responsible for the downregulation of *SCARB2*. In turn, the absence of regulation of this gene in the presence of zymosan, a representative of ß-glucan, suggests that other specific fungal epitopes might induce the expression of this gene during fungal infection, especially during *C. albicans* infection. In this study, other genes encoding lysosomal transmembrane proteins, *CD164* and *NPC1*, were analyzed. Croze et al. reported *CD164* encoding sialomucin protein (Endolyn-78) to be involved in the maturation of the endosomal-lysosomal compartment (Croze et al., 1989), while the Niemann-Pick disease type C1 (NPC1) protein encoded by the *NPC1* gene mediates intracellular cholesterol and sphingolipids trafficking into the late endosome and lysosome (Alam et al., 2012). *NPC1* is located in late endosomes and lysosomes and its encoded protein might promote the creation and/or movement of these compartments to and from the cell periphery (Ko et al., 2001). In our study, we have shown the up-regulation of *CD164* and *NPC1*in human monocytes specifically after fungal challenge, which again suggests the importance of biogenesis and functionality of the lysosome for fungal clearance in monocytes.

### Functional relevance of differentially expressed non-lysosome-related genes

Most of the genes further analyzed in this study associated to the proper biosynthesis and functionality of the lysosome during fungal infection. In addition, other mechanisms, such as immune cells recruitment, phagocytosis and nutrient metabolism, are also known to be crucial for a successful fungal killing and clearance by the phagocytes. Thus, other genes identified in this study to be fungal-challenge specific are involved in those pathways and might play an important role during fungal infection. For instance, *BAG3* encodes the BAG family molecular chaperone regulator 3 (BAG3) protein which regulates macroautophagy for degradation of polyubiquitinated proteins (Gamerdinger et al., 2009). The peroxisome proliferator-activated receptor gamma (*PPARG*) is a gene expressed in macrophages and its encoding a protein that plays a central role in regulating fatty acid storage and glucose metabolism (Tyagi et al., 2011). Fatty Acid Binding Protein 5 (FABP5) is a protein encoded by *FABP5* gene and plays a role in the uptake of fatty acids, transport phenomena and fatty acid metabolism (Moore et al., 2015). The *HMOX1* gene, encoding heme oxygenase-1 (HO-1), has been shown to be required for immune cell protection against systemic infections (Silva-Gomes et al., 2013). Primarily, HO-1 degrades heme into biliverdin and carbon monoxide (CO). CO has shown different effects, it supports anti-inflammatory cytokine expression (Piantadosi et al., 2011) but may in turn increase the virulence of the infection (Navarathna and Roberts, 2010). The C-C Chemokine Receptor 1 (CCR1), encoded by the *CCR1* gene, has been shown to be widely expressed in immune cells and it was associated with the maintenance of chemokine gradients during infection (Lionakis et al., 2012).

In summary, by integrating our combined classifier approach with distinct differential gene expression analysis across well selected, different studies investigating diverse species of pathogens, we could identify genes that are up-regulated in monocytes during fungal infection, much more or exclusively in comparison to a bacterial infection. Once fungi are phagocytosed, monocytes display transcriptional and translational reprogramming, adapting their physiology and killing mechanisms to fungal-derived stressors. In our study, we show the up-regulation of fungi-specific genes, which seem to be important in the fungal-derived reprogramming. Moreover, the application of the combined classifier approach made it possible, for the first time, to identify lysosome-related gene expression as a monocyte-specific footprint of fungal infections. Determining whether loss of the candidate genes have any functional impact on infection is also of great importance. siRNA-mediated knock-down experiments, combined with pathogen-challenge should be performed in the future. The multiple readouts with possible effects on phagocytosis, killing, cytokine production and metabolism would represent an attractive target for follow-up studies.

## Author contributions

JL and RK conceived and designed the study. MB and RC generated the Saraiva microarray dataset. CZ-B, ML, TK, and HS supported the analysis of the Klassert dataset and performed the RT-qPCR. JL performed all bioinformatics analysis. Analysis and interpretation of results was performed by JL, CZ-B, and TK. JL, CZ-B, TK, and RK wrote the manuscript. All authors have read and approved the final version of the manuscript.

### Conflict of interest statement

The authors declare that the research was conducted in the absence of any commercial or financial relationships that could be construed as a potential conflict of interest.

## References

[B1] Alam M. S. Getz M. Safeukui I. Yi S. Tamez P. Shin J.. (2012). Genomic expression analyses reveal lysosomal, innate immunity proteins, as disease correlates in murine models of a lysosomal storage disorder. PLoS ONE 7:e48273. 10.1371/journal.pone.0048273 23094108 PMC3477142

[B2] Allantaz F. Cheng D. T. Bergauer T. Ravindran P. Rossier M. F. Ebeling M.. (2012). Expression profiling of human immune cell subsets identifies miRNA-mRNA regulatory relationships correlated with cell type specific expression. PLoS ONE 7:e29979. 10.1371/journal.pone.0029979 22276136 PMC3262799

[B3] Batuwita R. Palade V. (2013). Class imbalance learning methods for support vector, in Imbalanced Learning: Foundations, Algorithms, Applications, eds He H. Ha Y. (Hoboken, NJ: John Wiley & Sons, Inc.), 83–100.

[B4] Benjamini Y. Hochberg Y. (1995). Controlling the false discovery rate - a practical and powerful approach to multiple testing. J. R. Stat. Soc. Ser.B Stat. Methodol. 57, 289–300.

[B5] Bloos F. Reinhart K. (2014). Rapid diagnosis of sepsis. Virulence 5, 154–160. 10.4161/viru.27393 24335467 PMC3916369

[B6] Brown M. P. Grundy W. N. Lin D. Cristianini N. Sugnet C. W. Furey T. S.. (2000). Knowledge-based analysis of microarray gene expression data by using support vector machines. Proc. Natl. Acad. Sci. U.S.A. 97, 262–267. 10.1073/pnas.97.1.262 10618406 PMC26651

[B7] Croze E. Ivanov I. E. Kreibich G. Adesnik M. Sabatini D. D. Rosenfeld M. G. (1989). Endolyn-78, a membrane glycoprotein present in morphologically diverse components of the endosomal and lysosomal compartments: implications for lysosome biogenesis. J. Cell Biol. 108, 1597–1613. 2654137 10.1083/jcb.108.5.1597 PMC2115562

[B8] Darmoise A. Teneberg S. Bouzonville L. Brady R. O. Beck M. Kaufmann S. H.. (2010). Lysosomal α-galactosidase controls the generation of self lipid antigens for natural killer T cells. Immunity 33, 216–228. 10.1016/j.immuni.2010.08.003 20727792 PMC4018304

[B9] De Francesco P. N. Mucci J. M. Ceci R. Fossati C. A. Rozenfeld P. A. (2013). Fabry disease peripheral blood immune cells release inflammatory cytokines: role of globotriaosylceramide. Mol Genet. Metabol. 109, 93–99. 10.1016/j.ymgme.2013.02.003 23452955

[B10] Didonna A. Cekanaviciute E. Oksenberg J. R. Baranzinia S. E. (2016). Immune cell-specific transcriptional profiling highlights distinct molecular pathways controlled by Tob1 upon experimental autoimmune encephalomyelitis. Sci. Rep. 6:31603. 10.1038/srep31603 27546286 PMC4992865

[B11] Du P. Kibbe W. A. Lin S. M. (2008). lumi: a pipeline for processing illumina microarray. Bioinformatics 24, 1547–1548. 10.1093/bioinformatics/btn224 18467348

[B12] Furey T. S. Cristianini N. Duffy N. Bednarski D. W. Schummer M. Haussler D. (2000). Support vector machine classification and validation of cancer tissue samples using microarray expression data. Bioinformatics 16, 906–914. 10.1093/bioinformatics/16.10.906 11120680

[B13] Gamerdinger M. Hajieva P. Kaya A. M. Wolfrum U. Hartl F. U. Behl C. (2009). Protein quality control during aging involves recruitment of the macroautophagy pathway by BAG3. EMBO J. 28, 889–901. 10.1038/emboj.2009.29 19229298 PMC2647772

[B14] Gardinassi L. G. Garcia G. R. Costa C. H. N. Silva V. C. de Miranda Santos I. K. F. (2016). Blood transcriptional profiling reveals immunological signatures of distinct states of infection of humans with leishmania infantum. PLOS Negl. Trop. Dis. 10:e0005123. 10.1371/journal.pntd.0005123 27828962 PMC5102635

[B15] Golub T. R. Slonim D. K. Tamayo P. Huard C. Gaasenbeek M. Mesirov J. P. (1999). Molecular classification of cancer: class discovery and class prediction by gene expression monitoring. Science 286, 531–537. 10521349 10.1126/science.286.5439.531

[B16] Gonzalez A. Valeiras M. Sidransky E. Tayebi N. (2014). Lysosomal integral membrane protein-2: a new player in lysosome-related pathology. Mol. Genet. Metabol. 111, 84–91. 10.1016/j.ymgme.2013.12.005 24389070 PMC3924958

[B17] Hessle C. C. Andersson B. Wold A. E. (2005). Gram-positive and Gram-negative bacteria elicit different patterns of pro-inflammatory cytokines in human monocytes. Cytokine 30, 311–318. 10.1016/j.cyto.2004.05.008 15935951

[B18] Huang da W. Lempicki R. A. Sherman B. T. (2009). Systematic and integrative analysis of large gene lists using DAVID bioinformatics resources. Nat. Prot. 4, 44–57. 10.1038/nprot.2008.211 19131956

[B19] Iwabuchi K. Nakayama H. Oizumi A. Suga Y. Ogawa H. Takamori K. (2015). Role of ceramide from glycosphingolipids and its metabolites in immunological and inflammatory responses in humans. Mediators Inflamm. 2015:120748. 10.1155/2015/120748 26609196 PMC4644562

[B20] Jimenez-Lucho V. Ginsburg V. Krivan H. C. (1990). *Cryptococcus neoformans, Candida albicans*, and other fungi bind specifically to the glycosphingolipid lactosylceramide (GAl beta 1-4Glc beta 1-1Cer), a possible adhesion receptor for yeasts. Infect. Immun. 58, 2085–2090. 2194958 10.1128/iai.58.7.2085-2090.1990 PMC258780

[B21] Kirn T. J. Weinstein M. P. (2013). Update on blood cultures: how to obtain, process, report, and interpret. Clin. Microbiol. Infect. 19, 513–520. 10.1111/1469-0691.12180 23490046

[B22] Klassert T. E. Bräuer J. Hölzer M. Stock M. Riege K. Zubiría-Barrera C.. (2017). Differential effects of vitamins A and D on the transcriptional landscape of human monocytes during infection. Sci. Rep. 7:40599. 10.1038/srep40599 28094291 PMC5240108

[B23] Klassert T. E. Hanisch A. Bräuer J. Klaile E. Heyl K. A. Mansour M. K.. (2014). Modulatory role of vitamin A on the Candida albicans-induced immune response in human monocytes. Med. Microbiol. Immunol. 203, 415–424. 10.1007/s00430-014-0351-4 25129478 PMC4232755

[B24] Ko D. C. Gordon M. D. Jin J. Y. Scott M. P. (2001). Dynamic movements of organelles containing Niemann-Pick C1 protein: NPC1 involvement in late endocytic events. Mol. Biol. Cell. 12, 601–614. 10.1091/mbc.12.3.601 11251074 PMC30967

[B25] Lauvau G. Loke P. Hohl T. M. (2015). Monocyte-mediated defense against bacteria, fungi, and parasites. Semin. Immunol. 27, 397–409. 10.1016/j.smim.2016.03.014 27021645 PMC4900144

[B26] Lee E. K. (2007). Large-scale optimization-based classification models in medicine and biology. Ann. Biomed. Eng. 35, 1095–1109. 10.1007/s10439-007-9317-7 17503186

[B27] Lionakis M. S. Fischer B. G. Lim J. K. Swamydas M. Wan W. Richard Lee C. C.. (2012). Chemokine receptor Ccr1 drives neutrophil-mediated kidney immunopathology and mortality in invasive candidiasis. PLoS Pathog. 8:e1002865. 10.1371/journal.ppat.1002865 22916017 PMC3420964

[B28] Liu Y. Shetty A. C. Schwartz J. A. Bradford L. L. Xu W. Phan Q. T. (2015). New signaling pathways govern the host response to *C. albicans* infection in various niches. Genome Res. 125, 679–689. 10.1101/gr.187427.114 PMC4417116 25858952

[B29] McDermott J. E. Wang J. Mitchell H. Webb-Robertson B. J. Hafen R. Ramey J.. (2012). Challenges in biomarker discovery: combining expert insights with statistical analysis of complex omics data. Expert Opin. Med. Diagn. 7, 1–15. 10.1517/17530059.2012.718329 23335946 PMC3548234

[B30] Moore S. M. Holt V. V. Malpass L. R. Hines I. N. Wheeler M. D. (2015). Fatty acid-binding protein 5 limits the anti-inflammatory response in murine macrophages. Mol. Immunol. 67, 265–275. 10.1016/j.molimm.2015.06.001 26105806 PMC4565774

[B31] Müller M. M. Lehmann R. Klassert T. E. Reifenstein S. Conrad T. Moore C.. (2017). Global analysis of glycoproteins identifies markers of endotoxin tolerant monocytes and GPR84 as a modulator of TNFα expression. Sci. Rep. 7:838. 10.1038/s41598-017-00828-y 28404994 PMC5429802

[B32] Navarathna D. H. Roberts D. D. (2010). *Candida albicans* heme oxygenase and its product CO contribute to pathogenesis of candidemia and alter systemic chemokine and cytokine expression. Free Radic. Biol. Med. 49, 1561–1573. 10.1016/j.freeradbiomed.2010.08.020 20800092 PMC2952735

[B33] Netea M. G. Brown G. D. Kullberg B. J. Gow N. A. (2008). An integrated model of the recognition of *Candida albicans* by the innate immune system. Nat. Rev. Microbiol. 6, 67–78. 10.1038/nrmicro1815 18079743

[B34] Ng H. H. Frantz C. E. Rausch L. Fairchild D. C. Shimon J. Riccio E.. (2005). Gene expression profiling of mouse host response to Listeria monocytogenes infection. Genomics 86, 657–667. 10.1016/j.ygeno.2005.07.005 16102935

[B35] Ngo L. Y. Kasahara S. Kumasaka D. K. Knoblaugh S. E. Jhingran A. Hohl T. M. (2014). Inflammatory monocytes mediate early and organ-specific innate defense during systemic candidiasis. J. Infect. Dis. 209, 109–119. 10.1093/infdis/jit413 23922372 PMC3864383

[B36] Noble W. S. (2004). Support vector machine applications in computational biology, in Kernel Methods in Computational Biology, eds Schölfkopf B. Tsuda K. Vert J. (Cambridge, MA: MIT Press), 71–92.

[B37] Palmer C. Diehn M. Alizadeh A. A. Brown P. O. (2006). Cell-type specific gene expression profiles of leukocytes in human peripheral blood. BMC Genomics 7:115. 10.1186/1471-2164-7-115 16704732 PMC1479811

[B38] Petryszak R. Keays M. Tang Y. A. Fonseca N. A. Barrera E. Burdett T.. (2016). Expression Atlas update - An integrated database of gene and protein expression in humans, animals and plants. Nucleic Acids Res. 44, D746–D752. 10.1093/nar/gkv1045 26481351 PMC4702781

[B39] Pfaffl M. W. Tichopad A. Prgomet C. Neuvians T. P. (2004). Determination of stable housekeeping genes, differentially regulated target genes and sample integrity: bestKeeper–excel-based tool using pair-wise correlations. Biotechnol. Lett. 26, 509–515. 10.1023/B:BILE.0000019559.84305.47 15127793

[B40] Piantadosi C. A. Withers C. M. Bartz R. R. MacGarvey N. C. Fu P. Sweeney T. E.. (2011). Heme oxygenase-1 couples activation of mitochondrial biogenesis to anti-inflammatory cytokine expression. J. Biol. Chem. 286, 16374–16385. 10.1074/jbc.M110.207738 21454555 PMC3091243

[B41] Riege K. Hölzer M. Klassert T. E. Barth E. Bräuer J. Collatz M.. (2017). Massive effect on LncRNAs in human monocytes during fungal and bacterial infections and in response to vitamins A and D. Sci. Rep. 7:40598. 10.1038/srep40598 28094339 PMC5240112

[B42] Rieu I. Powers S. J. (2009). Real-time quantitative RT-PCR - design, calculations, and statistics. Plant Cell 21, 1031–1103. 10.1105/tpc.109.066001 19395682 PMC2685626

[B43] Ritchie M. E. Phipson B. Wu D. Hu Y. Law C. W. Shi W.. (2015). limma powers differential expression analyses for RNA-sequencing and microarray studies. Nucleic Acids Res. 43:e47. 10.1093/nar/gkv007 25605792 PMC4402510

[B44] Saeys Y. Inza I. Larrañaga P. (2007). A review of feature selection techniques in bioinformatics. Bioinformatics 23, 2507–2517. 10.1093/bioinformatics/btm344 17720704

[B45] Saraiva J. P. Marcus O. Antje B. Cora A. Tilman K. Markus B. (2016). Integrating classifiers across datasets improves consistency of biomarker predictions for sepsis. In 6th IFAC Conference on Foundations of Systems Biology in Engineering. Magdeburg: Elsevier ScienceDirect.

[B46] Shi C. Pamer E. G. (2011). Monocyte recruitment during infection and inflammation. Nat. Rev. Immunol. 11, 762–774. 10.1038/nri3070 21984070 PMC3947780

[B47] Silva-Gomes S. Appelberg R. Larsen R. Soares M. P. Gomes M. S. (2013). Heme catabolism by heme oxygenase-1 confers host resistance to Mycobacterium infection. Infect. Immun. 81, 2536–2545. 10.1128/IAI.00251-13 23630967 PMC3697604

[B48] Smeekens S. P. Ng A. Kumar V. Johnson M. D. Plantinga T. S. van Diemen C.. (2013). Functional genomics identifies type I interferon pathway as central for host defense against *Candida albicans*. Nat. Commun. 4:1342. 10.1038/ncomms2343 23299892 PMC3625375

[B49] Tyagi S. Gupta P. Saini A. S. Kaushal C. Sharma S. (2011). The peroxisome proliferator-activated receptor: a family of nuclear receptors role in various diseases. J. Adv. Pharmaceut. Technol. Res. 2, 236–240. 10.4103/2231-4040.90879 22247890 PMC3255347

[B50] Wong K. L. Tai J. J. Wong W. C. Han H. Sem X. Yeap W. H.. (2011). Gene expression profiling reveals the defining features of the classical, intermediate, and nonclassical human monocyte subsets. Blood 118, e16–e31. 10.1182/blood-2010-12-326355 21653326

[B51] Yamayoshi S. Fujii K. Koike S. (2014). Receptors for enterovirus 71. Emerg. Microbes Infect. 3:e53. 10.1038/emi.2014.49 26038749 PMC4126179

[B52] Zuker M. (2003). Mfold web server for nucleic acid folding and hybridization prediction. Nucleic Acids Res. 31, 3406–3415. 10.1093/nar/gkg595 12824337 PMC169194

