# Supplementary material for: Combination of Classifiers Identifies Fungal-Specific Activation of Lysosome Genes in Human Monocytes
Source: Front Microbiol. 2017 Nov 29;8:2366. doi: 10.3389/fmicb.2017.02366 (PMC5712586; doi:10.3389/fmicb.2017.02366)
Supplement: Supplementary file 1 [file Table1.PDF]

Table S1 - Primer sequences for indicated genes employed in qRT-PCR

HUMAN GENE	SYMBOL	FORWARD PRIMER (5'->3')	REVERSE PRIMER (5'->3')	SIZE (BP)
**PEPTIDYL-PROLYL CIS-TRANS ISOMERASE B**	*PPIB*	ATGTAGGCCGGGTGATCTTT	TGAAGTTCTCATCGGGGAAG	219
**GALACTOSIDASE ALPHA**	*GLA*	AGGAAGAGCCAGATTCCTGC	GCGAATCCCATGAGGAAAGC	185
**SCAVENGER RECEPTOR CLASS B MEMBER 2**	*SCARB2*	GCATGCACCCAAATCAGGAA	GTCGACTCGCCGTCTCTTTA	210
**BCL2 ASSOCIATED ATHANOGENE 3**	*BAG3*	CCAGAAACCACTCAGCCAGA	CGGAATGGAGATGTACCCCC	204
**PEROXISOME PROLIFERATOR ACTIVATED RECEPTOR GAMMA**	*PPARG*	ACAGATCCAGTGGTTGCAGA	AGATGCAGGCTCCACTTTGA	81
**FATTY ACID BINDING PROTEIN 5**	*FABP5*	AAACCACAGCTGATGGCAGA	GCTTTCCTTCCCATCCCACT	92
**CLUSTER OF DIFFERENTIATION 164**	*CD164*	CCGAACGTGACGACTTTAGC	GAAGTCTGTCGTGTTCCCCA	234
**NPC1 - NPC INTRACELLULAR CHOLESTEROL TRANSPORTER 1**	*NPC1*	AGCCACATAACCAGAGCGTT	GAGTGGCTCCCAGTAAGACC	221
**HEME OXYGENASE 1**	*HMOX1*	AACTTTCAGAAGGGCCAGGT	AGACTGGGCTCTCCTTGTTG	115
**C-C MOTIF CHEMOKINE RECEPTOR 1**	*CCR1*	TCTTTGGGCTGGTATTGCCT	ACAGCCAGGTCCAAATGTCT	235
