# Supplementary material for: Combination of Classifiers Identifies Fungal-Specific Activation of Lysosome Genes in Human Monocytes
Source: Front Microbiol. 2017 Nov 29;8:2366. doi: 10.3389/fmicb.2017.02366 (PMC5712586; doi:10.3389/fmicb.2017.02366)
Supplement: Supplementary file 2 [file Table2.PDF]

Table S2 - Single and combined classifier gene lists ordered by highest presence in classifier combinations.

**Single classifier**	**# combinations**	**Combined classifier**	**# combinations**
C5AR1	2	*CCR1*	5
CCR1	2	*SCARB2*	5
CXCL10	2	*STK26*	5
EDN1	2	*EVL*	4
EVL	2	*GLA*	4
FXYD6	2	*LGALS9*	4
HMOX1	2	*SERPINA1*	4
SLC16A3	2	*SPP1*	4
SLFN12	2	*VAV1*	4
STK26	2	*ANXA1*	3
ACADVL	1	*BLMH*	3
ACVR1	1	*C1GALT1*	3
ADCY3	1	*CCL23*	3
ADORA2B	1	*CEP135*	3
ANAPC4	1	*CITED2*	3
ANXA1	1	*EDN1*	3
ARHGEF3	1	*FAM46A*	3
BCAR3	1	*FXYD6*	3
BTK	1	*HAVCR2*	3
C12orf10	1	*HMOX1*	3
C14orf159	1	*MOV10*	3
C1GALT1	1	*PCID2*	3
CCL20	1	*PELI1*	3
CCL23	1	*RHOU*	3
CCL5	1	*SATB1*	3
CCL8	1	*SLFN12*	3
CCR4	1	*SMCO4*	3
CCR7	1	*SOWAHC*	3
CD247	1	*ST3GAL5*	3

CD40	1	*TAGAP*	3
CD68	1	*TBC1D9*	3
CD86	1	*ACADVL*	2
CDCA4	1	*ARHGEF3*	2
CDK6	1	*BCAR3*	2
CEBPB	1	*C12orf10*	2
CEP135	1	*C14orf159*	2
CEP295	1	*CD40*	2
CH25H	1	*CD86*	2
CISD1	1	*CDK6*	2
CISH	1	*CLCF1*	2
CLCF1	1	*CLDN23*	2
CLDN23	1	*CTSC*	2
CMTM7	1	*DDX60L*	2
CRIPAK	1	*EMP1*	2
CRYGS	1	*ENC1*	2
CXCL1	1	*ETV3*	2
CXCL11	1	*GEM*	2
CXCL2	1	*GRAMD1A*	2
CXCL3	1	*IFNB1*	2
CXCL5	1	*IVNS1ABP*	2
CXCL6	1	*JUN*	2
CXCL8	1	*KLF4*	2
CXCL9	1	*MGAT2*	2
CXCR4	1	*NCOA7*	2
CXCR6	1	*PAPSS1*	2
DDX60L	1	*PARP10*	2
DHRS9	1	*RBCK1*	2
EGR2	1	*RGS1*	2
EIF2AK3	1	*SEPT6*	2
EIF2AK4	1	*SLC16A3*	2
ENC1	1	*SLC7A7*	2
ETV3	1	*SP140*	2

FAM46A	1	*SPRY2*	2
FPR2	1	*TNFRSF1B*	2
FPR3	1	*TNFRSF9*	2
GBP3	1	*TNFSF10*	2
GEM	1	*TRIP10*	2
GLA	1	*UNC93B1*	2
GLIPR2	1	*WIPF1*	2
GNAQ	1	*ACVR1*	1
GNG2	1	*ANAPC4*	1
GPAT3	1	*APOBEC3A*	1
GPR18	1	*ATP6V0A1*	1
GRAMD1A	1	*BATF2*	1
HACD3	1	*BLVRA*	1
HAVCR2	1	*C5AR1*	1
HCAR2	1	*CCL8*	1
HDAC1	1	*CD14*	1
HK2	1	*CD68*	1
HSPBAP1	1	*CDCA4*	1
IFNB1	1	*CEBPB*	1
IGF2R	1	*CENPW*	1
IL12A	1	*CEP295*	1
IL27RA	1	*CHMP5*	1
IQSEC1	1	*CISD1*	1
IRF2	1	*CISH*	1
IVNS1ABP	1	*CLEC5A*	1
JAK2	1	*CMTM7*	1
KLF4	1	*CRIPAK*	1
KLHL21	1	*CRYGS*	1
LGALS9	1	*CUL4A*	1
MAP3K1	1	*CXCL10*	1
MASTL	1	*CXXC5*	1
MGAT2	1	*DDX60*	1
MOV10	1	*DHRS9*	1

MYC	1	*DHX58*	1
NAGK	1	*EGR2*	1
NCF1	1	*EPB41L3*	1
NCF1C	1	*FNDC3A*	1
NCOA7	1	*GLIPR2*	1
NDUFAF7	1	*GPAT3*	1
NDUFV1	1	*GRHPR*	1
NPEPL1	1	*HACD3*	1
NSMAF	1	*HCAR2*	1
NUB1	1	*HK2*	1
PAPSS1	1	*HSPA6*	1
PARP1	1	*HSPB1*	1
PARP10	1	*HSPBAP1*	1
PARP4	1	*IGF2R*	1
PCID2	1	*IL27RA*	1
PCNT	1	*IQSEC1*	1
PELI1	1	*IRF2*	1
PIK3CB	1	*MICAL1*	1
PIK3CG	1	*MRPS24*	1
PLA2G7	1	*MYD88*	1
PLCXD1	1	*NCF1*	1
PPBP	1	*NCF1C*	1
PRKAG2	1	*NDUFAF7*	1
RAB3IP	1	*NDUFV1*	1
RABGAP1L	1	*NISCH*	1
RBCK1	1	*NSMAF*	1
RGS1	1	*NUB1*	1
RHBDD2	1	*ORC2*	1
RNF144B	1	*PARP4*	1
RPAP2	1	*PCNT*	1
RPUSD2	1	*PGD*	1
S100A9	1	*PLCXD1*	1
S1PR4	1	*PPM1M*	1

SATB1	1	*PRKAG2*	1
SCARB2	1	*RABGAP1L*	1
SDSL	1	*RASGRP3*	1
SEPT6	1	*RHBDD2*	1
SERPINA1	1	*RHOH*	1
SLC7A7	1	*RNF144B*	1
SMAD3	1	*RPAP2*	1
SMCHD1	1	*S100A9*	1
SMCO4	1	*SDSL*	1
SOWAHC	1	*SP100*	1
SP100	1	*ST6GALNAC6*	1
SP140	1	*STAP1*	1
SPRY2	1	*STAT2*	1
ST3GAL5	1	*STAT5A*	1
STAP1	1	*TGFBI*	1
STAT5A	1	*TMEM106A*	1
SUCNR1	1	*TMEM243*	1
SYNJ2BP	1	*TNFAIP2*	1
TAGAP	1	*TNFSF13B*	1
TBC1D9	1	*TPMT*	1
TGFBI	1	*TRAFD1*	1
THOC1	1	*TRANK1*	1
TMEM243	1	*TRIB2*	1
TNFAIP2	1	*TRIM21*	1
TNFRSF1B	1	*TSC22D1*	1
TNFSF10	1	*TTC14*	1
TNFSF13B	1	*TTYH3*	1
TNFSF15	1	*UBA7*	1
TP53INP2	1	*UBASH3B*	1
TPMT	1	*USP11*	1
TRAFD1	1	*VMO1*	1
TRANK1	1	*WDFY2*	1
TRIB2	1	*XRN1*	1

TRIM21	1	*ZBTB32*	1
TRIM5	1	*ZNF786*	1
TRIP10	1	*ZRSR2*	1
TTC14	1		
UBA7	1		
UBASH3B	1		
UNC93B1	1		
USP18	1		
VAV1	1		
VAV3	1		
VMO1	1		
WDFY2	1		
ZNF700	1		
ZRSR2	1		
