# Supplementary material for: Combination of Classifiers Identifies Fungal-Specific Activation of Lysosome Genes in Human Monocytes
Source: Front Microbiol. 2017 Nov 29;8:2366. doi: 10.3389/fmicb.2017.02366 (PMC5712586; doi:10.3389/fmicb.2017.02366)
Supplement: Supplementary file 3 [file Table3.PDF]

Table S3 - Enriched gene sets using PBMC-specific differentially expressed genes and up regulated in fungal *versus* bacterial induced immune cell response.

| Gene set | P-value |
|---|---|
| Jak-STAT signaling | 4.8E-4 |
| Cytokine-cytokine receptor interaction | 0.026 |
| Toll-like receptor signaling | 0.026 |
