# Supplementary material for: Combination of Classifiers Identifies Fungal-Specific Activation of Lysosome Genes in Human Monocytes
Source: Front Microbiol. 2017 Nov 29;8:2366. doi: 10.3389/fmicb.2017.02366 (PMC5712586; doi:10.3389/fmicb.2017.02366)
Supplement: Supplementary file 4 [file Table4.PDF]

Table S4 - RT-qPCR mean expression values across conditions and corresponding p-values for all genes of interest.

GENE	BONFERRONI'S MULTIPLE COMPARISONS TEST	MEAN DIFF,	95,00% CI OF DIFF,	SIGNIFICANT?	SUMMARY	ADJUSTED P VALUE
GLA	Ctrl vs. C.a.	-4,589	-5,368 to -3,81	Yes	****	<0,0001
	Ctrl vs. Asp.	-3,771	-4,55 to -2,992	Yes	****	<0,0001
	Ctrl vs. E.coli	-2,881	-3,66 to -2,102	Yes	****	<0,0001
	C.a. vs. Asp.	0,8175	0,03874 to 1,596	Yes	*	0,0384
	C.a. vs. E.coli	1,708	0,9287 to 2,486	Yes	***	0,0003
	Asp. vs. E.coli	0,89	0,1112 to 1,669	Yes	*	0,0236
SCARB2	Ctrl vs. C.a.	-0,5375	-1,074 to -0,001495	Yes	*	0,0493
	Ctrl vs. Asp.	0,0875	-0,4485 to 0,6235	No	ns	>0,9999
	Ctrl vs. E.coli	1,735	1,199 to 2,271	Yes	****	<0,0001
	C.a. vs. Asp.	0,625	0,089 to 1,161	Yes	*	0,021
	C.a. vs. E.coli	2,273	1,736 to 2,809	Yes	****	<0,0001
	Asp. vs. E.coli	1,648	1,111 to 2,184	Yes	****	<0,0001
PPARG	Ctrl vs. C.a.	-1,931	-3,035 to -0,8278	Yes	**	0,0014
	Ctrl vs. Asp.	-2,455	-3,558 to -1,352	Yes	***	0,0002
	Ctrl vs. E.coli	0,8488	-0,2547 to 1,952	No	ns	0,1759
	C.a. vs. Asp.	-0,5238	-1,627 to 0,5797	No	ns	0,8686
	C.a. vs. E.coli	2,78	1,677 to 3,883	Yes	****	<0,0001
	Asp. vs. E.coli	3,304	2,2 to 4,407	Yes	****	<0,0001
CD164	Ctrl vs. C.a.	-1,015	-1,58 to -0,4504	Yes	**	0,0011
	Ctrl vs. Asp.	-0,5775	-1,142 to -0,01295	Yes	*	0,0442
	Ctrl vs. E.coli	0,4813	-0,0833 to 1,046	No	ns	0,1113
	C.a. vs. Asp.	0,4375	-0,1271 to 1,002	No	ns	0,1704
	C.a. vs. E.coli	1,496	0,9317 to 2,061	Yes	****	<0,0001
	Asp. vs. E.coli	1,059	0,4942 to 1,623	Yes	***	0,0008
FABP5	Ctrl vs. C.a.	-1,405	-2,47 to -0,34	Yes	**	0,0098
	Ctrl vs. Asp.	-2,679	-3,744 to -1,614	Yes	****	<0,0001
	Ctrl vs. E.coli	0,575	-0,49 to 1,64	No	ns	0,6162
	C.a. vs. Asp.	-1,274	-2,339 to -0,2087	Yes	*	0,018
	C.a. vs. E.coli	1,98	0,915 to 3,045	Yes	***	0,0009
	Asp. vs. E.coli	3,254	2,189 to 4,319	Yes	****	<0,0001
BAG3	Ctrl vs. C.a.	-2,763	-3,418 to -2,107	Yes	****	<0,0001
	Ctrl vs. Asp.	-2,005	-2,66 to -1,35	Yes	****	<0,0001
	Ctrl vs. E.coli	-0,605	-1,26 to 0,05025	No	ns	0,0756
	C.a. vs. Asp.	0,7575	0,1023 to 1,413	Yes	*	0,0221
	C.a. vs. E.coli	2,158	1,502 to 2,813	Yes	****	<0,0001
	Asp. vs. E.coli	1,4	0,7448 to 2,055	Yes	***	0,0003

NPC1	Ctrl vs. C.a.	-0,8438	-1,571 to -0,1168	Yes	*	0,0216
	Ctrl vs. Asp.	-2,191	-2,918 to -1,464	Yes	****	<0,0001
	Ctrl vs. E.coli	-0,67	-1,397 to 0,05697	No	ns	0,0763
	C.a. vs. Asp.	-1,348	-2,074 to -0,6205	Yes	***	0,0009
	C.a. vs. E.coli	0,1738	-0,5532 to 0,9007	No	ns	>0,9999
	Asp. vs. E.coli	1,521	0,7943 to 2,248	Yes	***	0,0004
HMOX1	Ctrl vs. Asp.	-3,239	-4,228 to -2,249	Yes	****	<0,0001
	Ctrl vs. E.coli	3,406	2,417 to 4,396	Yes	****	<0,0001
	C.a. vs. Asp.	-3,165	-4,155 to -2,175	Yes	****	<0,0001
	C.a. vs. E.coli	3,48	2,49 to 4,47	Yes	****	<0,0001
	Asp. vs. E.coli	6,645	5,655 to 7,635	Yes	****	<0,0001
CCR1	Ctrl vs. C.a.	-1,114	-1,903 to -0,3249	Yes	**	0,0063
	Ctrl vs. Asp.	-1,198	-1,986 to -0,4086	Yes	**	0,0038
	Ctrl vs. E.coli	2,29	1,501 to 3,079	Yes	****	<0,0001
	C.a. vs. Asp.	-0,08375	-0,8726 to 0,7051	No	ns	>0,9999
	C.a. vs. E.coli	3,404	2,615 to 4,193	Yes	****	<0,0001
	Asp. vs. E.coli	3,488	2,699 to 4,276	Yes	****	<0,0001
