# Supplementary material for: Combination of Classifiers Identifies Fungal-Specific Activation of Lysosome Genes in Human Monocytes
Source: Front Microbiol. 2017 Nov 29;8:2366. doi: 10.3389/fmicb.2017.02366 (PMC5712586; doi:10.3389/fmicb.2017.02366)
Supplement: Supplementary file 5 [file Image1.PDF]

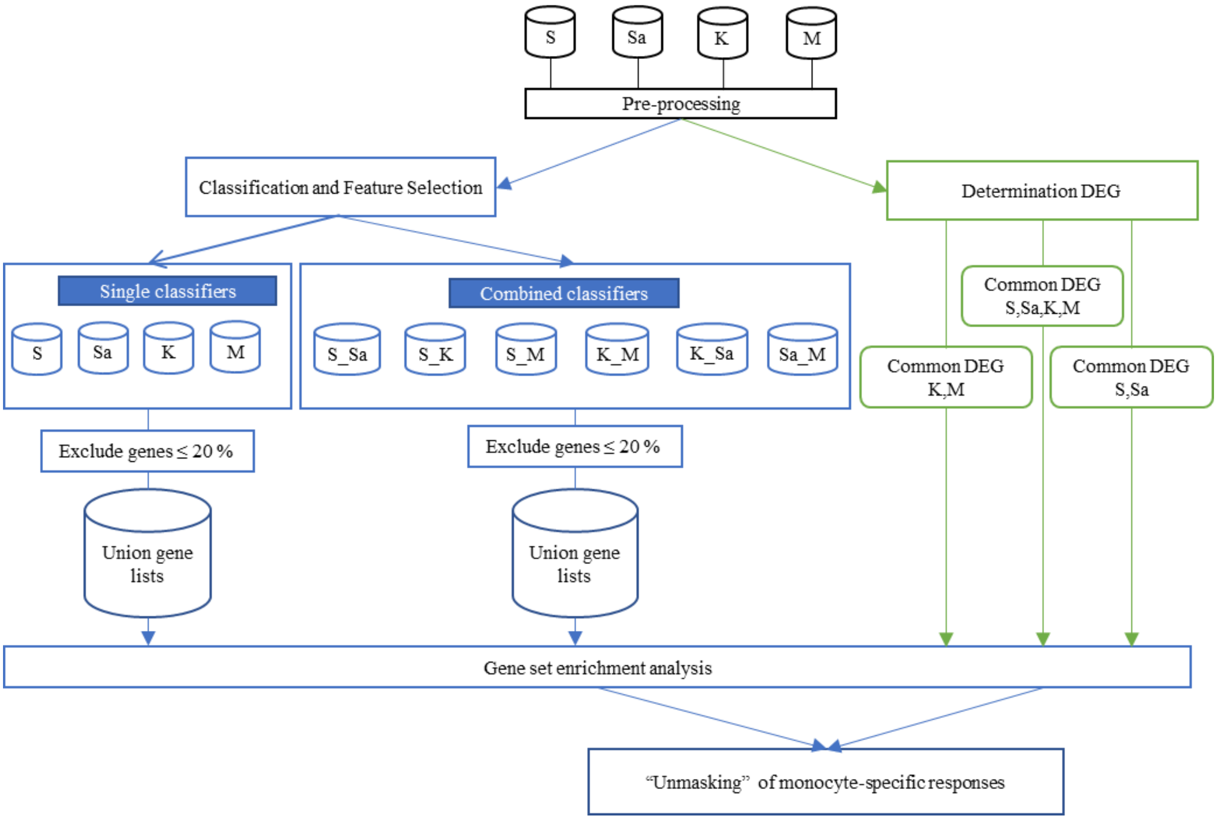

Supplementary Figure S1: Workflow for generating gene signatures from single and combined classifiers and determination of differentially expressed genes. Gene set enrichment tests are then performed for all lists of resulting genes (single and combined classifiers, and consistently differentially expressed genes in: all datasets, only in monocyte stimulated datasets and in PBMC stimulated datasets.
