# Supplementary figures and images for: Combination of Classifiers Identifies Fungal-Specific Activation of Lysosome Genes in Human Monocytes
Source: Front Microbiol. 2017 Nov 29;8:2366. doi: 10.3389/fmicb.2017.02366 (PMC5712586; doi:10.3389/fmicb.2017.02366)

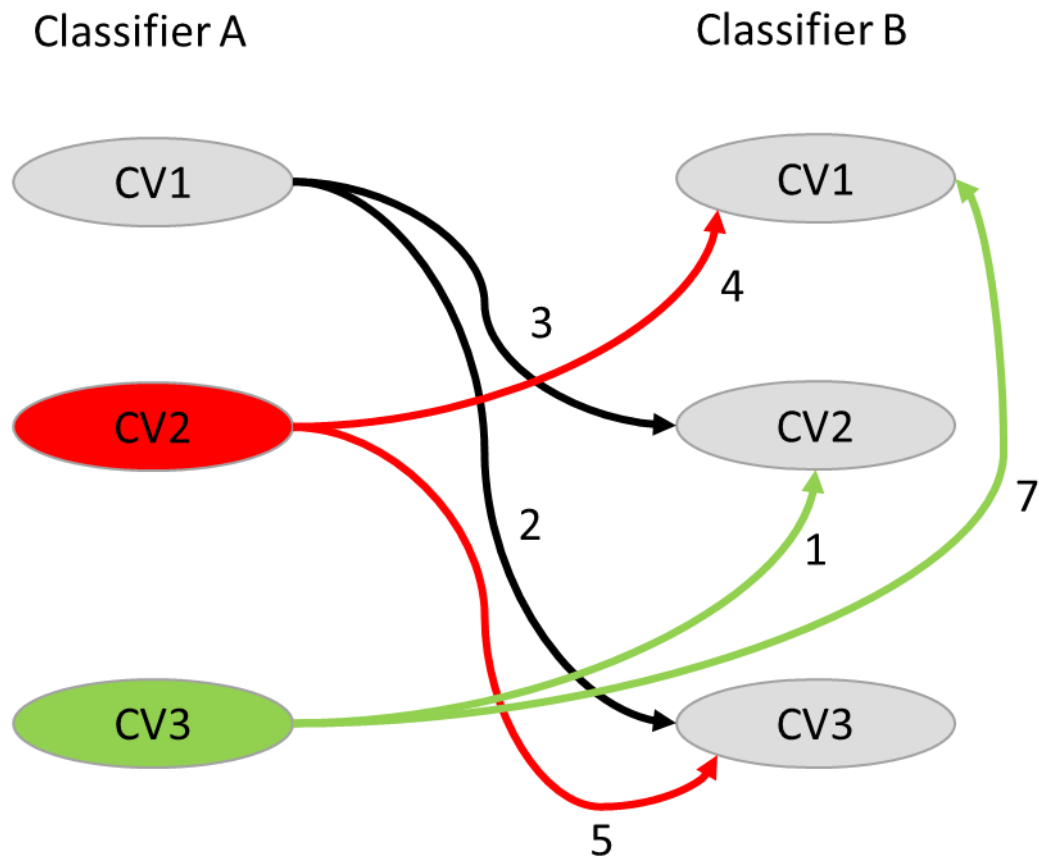

$$\text{Averaged POL} = (5+9+8)/6 = 3.66$$

Supplementary Figure S2: Pairwise overlap of cross validations.

Supplement: Supplementary file 6 [file Image2.PDF]
