# Supplementary material for: Combination of Classifiers Identifies Fungal-Specific Activation of Lysosome Genes in Human Monocytes
Source: Front Microbiol. 2017 Nov 29;8:2366. doi: 10.3389/fmicb.2017.02366 (PMC5712586; doi:10.3389/fmicb.2017.02366)
Supplement: Supplementary file 7 [file Image3.PDF]

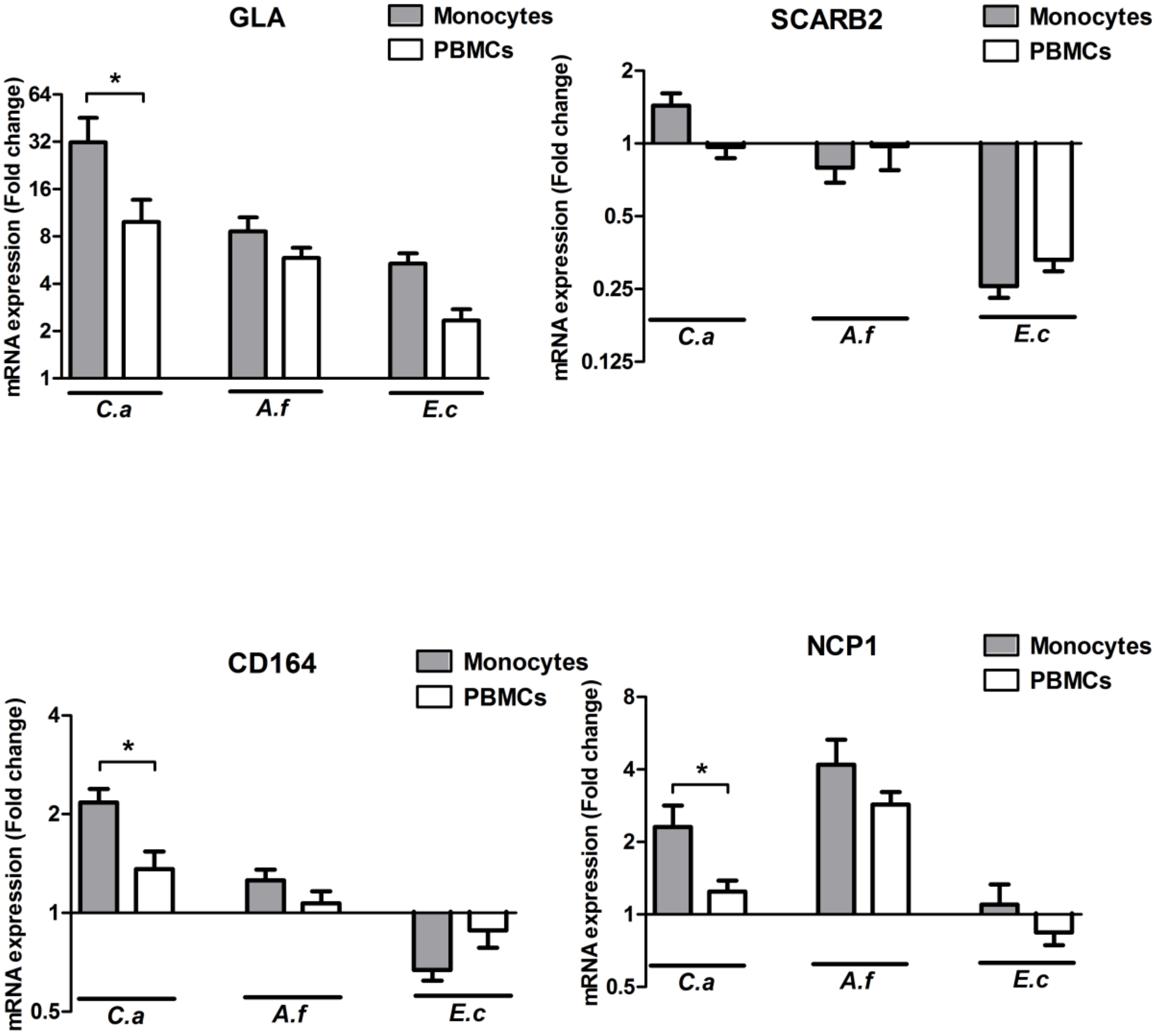

Supplementary Figure S3: Comparison of lysosomal-related gene expression levels in pathogen-stimulated PBMCs and monocytes. The validation experiments were repeated with 4 additional donors, from which both monocyte and PBMC fractions were isolated. These were then separately and simultaneously stimulated with *C. albicans* (C.a.), *A. fumigatus* (A.f.) and *E. coli* (E.c.) as detailed in the material and methods section. Results are presented as mean ± SE of the fold change relative to the control (unstimulated cells). Shown is also the statistical significance after repeated measures Two-Way ANOVA with Bonferroni post-hoc test (*p<0.05).
