# Supplementary material for: Combination of Classifiers Identifies Fungal-Specific Activation of Lysosome Genes in Human Monocytes
Source: Front Microbiol. 2017 Nov 29;8:2366. doi: 10.3389/fmicb.2017.02366 (PMC5712586; doi:10.3389/fmicb.2017.02366)
Supplement: Supplementary file 8 [file Image4.PDF]

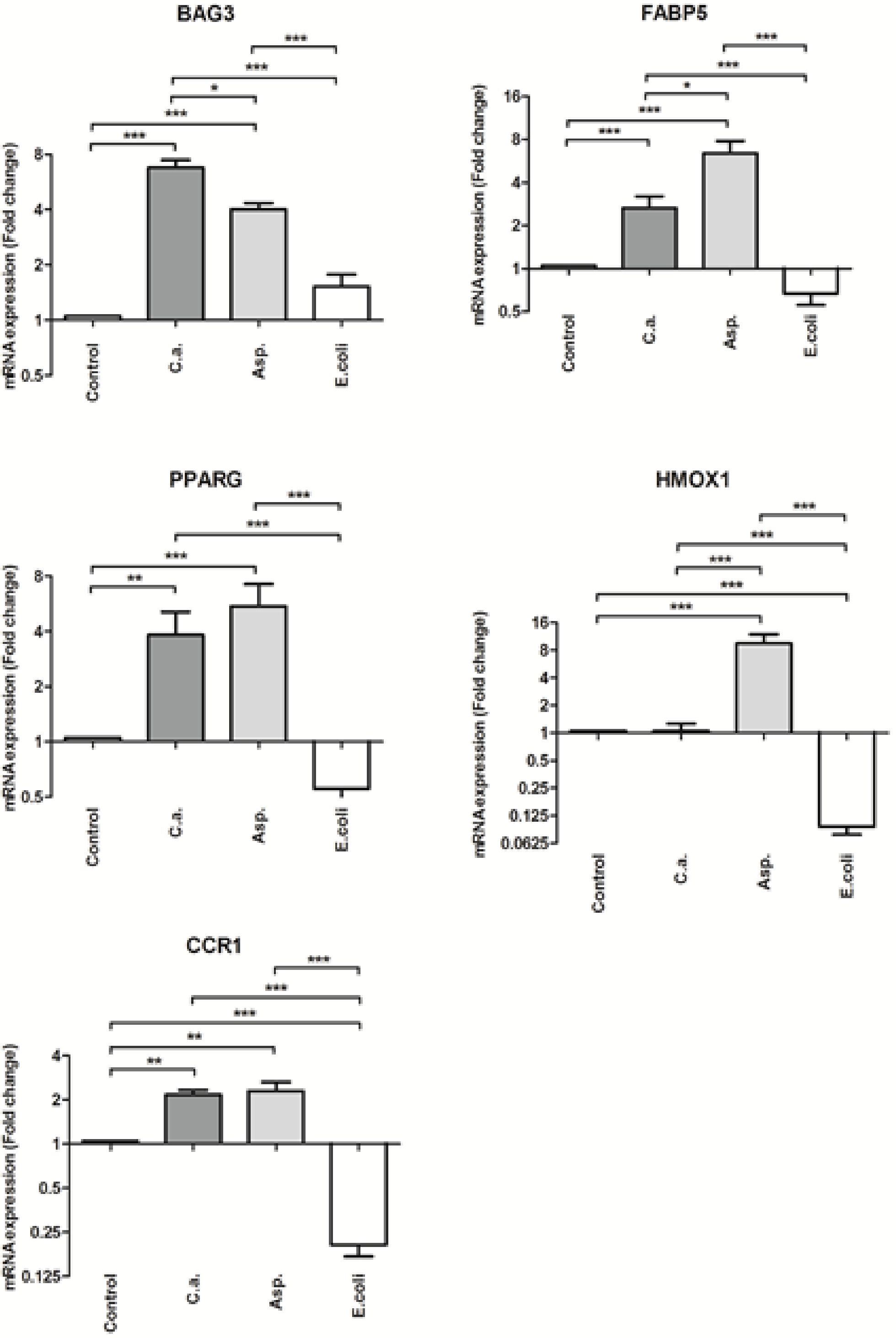

Figure S4: Relative mRNA expression of *BAG3*, *FABP5*, *PPARG*, *HMOX1* and *CCR1* after stimulation with *Candida albicans* (C.a.), *Aspergillus fumigatus* (Asp.) and *Escherichia coli* (E. coli). Data were obtained from four independent experiments, each performed with cells from different donors. Results are presented as mean ± SE of the fold change relative to the control (unstimulated cells). Shown is also the statistical significance after repeated measures One-Way ANOVA with Bonferroni correction (***$p<0.001$; **$p<0.01$; *$p<0.05$).
