# Supplementary material for: Combination of Classifiers Identifies Fungal-Specific Activation of Lysosome Genes in Human Monocytes
Source: Front Microbiol. 2017 Nov 29;8:2366. doi: 10.3389/fmicb.2017.02366 (PMC5712586; doi:10.3389/fmicb.2017.02366)
Supplement: Supplementary file 9 [file Image5.PDF]

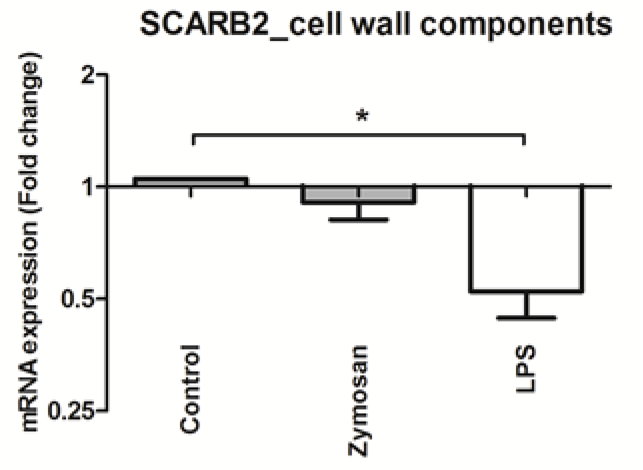

Supplementary Figure S5: Relative mRNA expression of SCARB2 after stimulation with Zymosan (1µg/ml) and LPS (50 ng/ml). Data were obtained from four independent experiments, each performed with cells from different donors. Results are presented as mean ± SE of the fold change relative to the control (unstimulated cells). Shown is also the statistical significance after repeated measures One-Way ANOVA with Dunnett post-hoc test (*$p<0.05$).
