# Supplementary material for: Combination of Classifiers Identifies Fungal-Specific Activation of Lysosome Genes in Human Monocytes
Source: Front Microbiol. 2017 Nov 29;8:2366. doi: 10.3389/fmicb.2017.02366 (PMC5712586; doi:10.3389/fmicb.2017.02366)
Supplement: Supplementary file 10 [file DataSheet1.PDF]

Text S1. Support Vector Machines (SVMs) implemented with Mixed Integer Linear Programming

The objective function was defined as the maximization of the margin of the SVM as seen in Eq. 1,

$$obj_{classifier} = max(t_1 - t_2) \qquad (1)$$

with t_1 and t_2 are the margins of class 1 and class 2 to the separating hyperplane, respectively. The objective function was subjected to the following constraints:

$$\sum_{i=1}^{nGenes} n_i g_{ij} \geq t_1 - My_j \;\; \forall j \in C_1 \qquad (2)$$

$$\sum_{i=1}^{nGenes} n_i g_{ij} \leq t_2 + My_j \;\; \forall j \in C_2 \qquad (3)$$

Equations 2 and 3 define the constraints applied to the classifier, for both class 1 (C_1) and class 2 (C_2), respectively. The scalar product of the gene expression of sample j with the weight n (for all genes $i \in \{1, \ldots, nGenes\}$) assigned them to a specific side of the margin but only for samples whose variables $y_j \in \{0,1\}$ were equal to 1. If this scalar product was less or equal than t_2 the samples were classified as group 2 and if greater or equal to t_1, classified as group 1. M was a large constant ("big M") that was set to allow exceptions if y_j equaled 1. Equation 4,

$$\sum_{j=1}^{nSamples} y_j \leq k \qquad (4)$$

constrained the number of allowed misclassifications k during the training (with nSamples training samples) of the classifier. k was set to 10% of the total number of samples $|S|$. To ensure that only genes i whose corresponding variables $x_i \in \{0,1\}$ equaled to 1 were used for classification, constraints of eq. 5 and 6 were established,

$$n_i \leq x_i \, \forall \, i \in G \qquad (5)$$

$$-n_i \leq x_i \, \forall \, i \in G \qquad (6)$$

The number of features (genes) which should be determined was constrained by equation 7, in our present study *l* was set to 30,

$$\sum_{i=1}^{nSamples} x_i \leq l \qquad (7)$$

x and *y* were defined as binary variables which belong to the set of genes *G* and samples *S* by:

$$x_i \in \{0,1\} \, \forall \, i \in G \qquad (8)$$

$$y_j \in \{0,1\} \, \forall \, j \in S \qquad (9)$$

To note, applying these sets of constraints generated a MILP problem and not an ordinary Linear Programming (LP) problem. Selection of consistent genes across all datasets required the combination of two independent MILPs. Each independent classifier was established by applying all previously defined equations. Next, the problems were connected by a combined objective function, Equation 10, adding up the objective functions of each classifier.

$$obj_{combined} = sum\left(obj_{classifier\ 1} + obj_{classifier\ 2}\right) \qquad (10)$$
